# Surface-enhanced laser desorption/ionization time-of-flight proteomic profiling of breast carcinomas identifies clinicopathologically relevant groups of patients similar to previously defined clusters from cDNA expression

**DOI:** 10.1186/bcr2101

**Published:** 2008-05-29

**Authors:** Kristyna Brozkova, Eva Budinska, Pavel Bouchal, Lenka Hernychova, Dana Knoflickova, Dalibor Valik, Rostislav Vyzula, Borivoj Vojtesek, Rudolf Nenutil

**Affiliations:** 1 Masaryk Memorial Cancer Institute, Zluty kopec 7, 656 53 Brno, Czech Republic; 2 Institute of Biostatistics and Analyses, Masaryk University, Kamenice 126/3, 625 00 Brno, Czech Republic; 3 Institute of Biochemistry, Faculty of Science, Masaryk University, Kotlarska 2, 611 37 Brno, Czech Republic; 4 Institute of Molecular Pathology, Faculty of Military Health Sciences, University of Defence, Trebesska 1575, 500 01 Hradec Kralove, Czech Republic

## Abstract

**Introduction:**

Microarray-based gene expression profiling represents a major breakthrough for understanding the molecular complexity of breast cancer. cDNA expression profiles cannot detect changes in activities that arise from post-translational modifications, however, and therefore do not provide a complete picture of all biologically important changes that occur in tumors. Additional opportunities to identify and/or validate molecular signatures of breast carcinomas are provided by proteomic approaches. Surface-enhanced laser desorption/ionization time-of-flight mass spectrometry (SELDI-TOF MS) offers high-throughput protein profiling, leading to extraction of protein array data, calling for effective and appropriate use of bioinformatics and statistical tools.

**Methods:**

Whole tissue lysates of 105 breast carcinomas were analyzed on IMAC 30 ProteinChip Arrays (Bio-Rad, Hercules, CA, USA) using the ProteinChip Reader Model PBS IIc (Bio-Rad) and Ciphergen ProteinChip software (Bio-Rad, Hercules, CA, USA). Cluster analysis of protein spectra was performed to identify protein patterns potentially related to established clinicopathological variables and/or tumor markers.

**Results:**

Unsupervised hierarchical clustering of 130 peaks detected in spectra from breast cancer tissue lysates provided six clusters of peaks and five groups of patients differing significantly in tumor type, nuclear grade, presence of hormonal receptors, mucin 1 and cytokeratin 5/6 or cytokeratin 14. These tumor groups resembled closely luminal types A and B, basal and HER2-like carcinomas.

**Conclusion:**

Our results show similar clustering of tumors to those provided by cDNA expression profiles of breast carcinomas. This fact testifies the validity of the SELDI-TOF MS proteomic approach in such a type of study. As SELDI-TOF MS provides different information from cDNA expression profiles, the results suggest the technique's potential to supplement and expand our knowledge of breast cancer, to identify novel biomarkers and to produce clinically useful classifications of breast carcinomas.

## Introduction

Extensive progress has been achieved towards understanding the epidemiology, clinical course, and basic biology of breast cancer. Several clinicopathologic factors – such as tumor grade, anatomical extent, presence/absence of lymph node metastases, presence of hormonal receptors and HER2/*neu *oncogene amplification – have been recognized as having prognostic and predictive value, influencing the management of patients suffering from breast cancer.

Microarray-based gene expression profiling represents another major breakthrough in the understanding of the molecular complexity of breast cancer [1,2]. Gene expression signatures have been identified that are associated with the presence of hormonal receptors, tumor grade and ability to metastasize [3-6]. These approaches can also identify gene expression signatures that predict response to specific chemotherapies or hormone-based therapies [7,8]. cDNA expression profiles cannot detect changes in activities that arise from post-translational modifications, however, and therefore do not provide a complete picture of all biologically important changes that occur in tumors.

Additional opportunities to identify and/or validate molecular signatures of breast carcinomas are provided by high-throughput proteomic approaches. Tissue microarrays represent the most developed high-throughput proteomic technology used to refine our knowledge of breast carcinoma. Immunohistochemical studies in tissue microarrays have confirmed the results of cDNA expression profiling and have identified identical breast carcinoma phenotypes; that is, two hormonal receptor-positive groups with luminal epithelial differentiation, a group with dominant HER2/*neu *expression, and a group with basal epithelial characteristics [9].

Hierarchical clustering of protein profiles obtained by immunohistochemistry also exhibits prognostic significance [10]. As immunohistochemical studies are able to evaluate only those proteins already described, another approach is necessary to identify novel proteins not yet associated with tumor clinicopathological characteristics. Surface-enhanced laser desorption/ionization time-of-flight mass spectrometry (SELDI-TOF MS) represents a high-throughput proteomic platform suitable for these types of study. SELDI-TOF MS is based on the surface capture of proteins or peptides from a biologic sample using defined chemical interactions with a solid surface [11]. The specific detection of ionized protein molecules is based on time-of-flight mass spectrometry.

The development of SELDI-TOF MS has overcome limitations of other proteomic approaches in terms of the inability to analyze hundreds of samples within a short time [12], which is essential for obtaining biologically and statistically relevant data in medical proteomic research. In addition, SELDI-TOF MS requires several times less starting material in comparison with two-dimensional polyacrylamide gel electrophoresis [13]. SELDI-TOF MS thus offers high-throughput protein profiling, leading to extraction of protein array data, which are often characterized by a large number of variables (the mass peaks), calling for effective and appropriate use of bioinformatics and statistical tools.

SELDI-TOF MS has been used to generate protein profiles of several cancer types, including breast cancer, to discriminate between malignant tumors and nonmalignant tumors with good sensitivity and specificity [14-17]. The majority of studies have analyzed body fluid samples such as serum [18], nipple aspirate fluid [14,19], or ductal lavage fluid [20]. Ricolleau and colleagues detected two prognostic biomarkers, ubiquitin and ferritin light chain, in node-negative breast cancer tumors [21]. Nakagawa and colleagues identified differences in the protein profiles of microdissected primary breast cancer tissue samples with and without axillary lymph node metastasis [17].

The aim of the present study was to evaluate tissue lysates of breast cancers by SELDI-TOF MS to identify protein patterns related to clinicopathological variables and/or tumor markers. To reveal similar protein expression profiles within 105 patients, unsupervised hierarchical clustering with a distance measure based on Spearman correlation and the Ward method of linkage of clusters was applied both to protein patterns (to reveal subgroups of patients) and to peaks (to reveal groups of peaks). The data show that this high-throughput protein profiling technique identifies patterns of expression that discriminate different types of breast tumors that group according to clinicopathological variables, and provides similar classification groups to cDNA expression profiling.

## Materials and methods

### Patient selection and characterization of specimens

Breast carcinomas of 105 female patients treated at the Masaryk Memorial Cancer Institute (Brno, Czech Republic) in 2004 and 2005 were collected from surgically treated women without neoadjuvant treatment and clinically documented distant metastases. Informed consent confirming the availability of redundant tissue samples for research use was obtained from each participating subject.

The main clinicopathological variables – including tumor type, grade and nuclear grade according to Elston and Ellis [22], and estrogen receptor (ER), progesterone receptor and HER2/*neu *status – were extracted from pathological records. Additional immunohistochemistry and fluorescence *in situ *hybridization were performed in tissue microarrays to estimate cyclin D_1 _overexpression and amplification, the presence of cytokeratin 5/6 and cytokeratin 14, mucin 1 and gross cystic fluid protein.

Information about the antibodies, probes and protocols applied is presented in Table 1. In our set of samples, the pT2 (57 cases, 54%) and pT3 (6 cases, 6%) tumors predominated above pT1 tumors (42 cases, 40%) because larger tumors were preferred due to availability of redundant tissue aliquots in the tissue bank. Consequently, metastasis in one or more axillary lymph node was seen in 64 cases (61%). The distribution in accordance with grade was uniform, with a slight predominance of moderately and poorly differentiated tumors (grade 1, 28%; grade 2, 35%; grade 3, 37%). The average age of the patients was identical to their median age (59 years). The series contained 66 ductal carcinomas, 22 lobular carcinomas, five atypical medullary carcinomas, two metaplastic carcinomas, three mucinous carcinomas, two papillary carcinomas, and five mixed ductal and lobular carcinomas. ER expression was positive in 85 cases (81%) and progesterone receptor expression was positive in 81 cases (77%). Initial screening for overexpression of HER2/*neu *by immunohistochemistry identified 20 cases (19%) with strong membrane staining (2+ or 3+). Gene amplification was subsequently validated using fluorescence *in situ *hybridization in 14 of these cases (13%).

**Table 1:** Antibodies and probes used

Immunohistochemistry	Manufacturer, clone	Antigen retrieval	Dilution	Evaluation
Estrogen receptor alpha	Lab Vision, SP1	0.01 M citrate NaOH, pH 6.0	1:4,000	Threshold at 5% positive nuclei
Estrogen receptor beta	Novocastra, EMR02	0.001 M EDTA-NaOH, pH 8.0	1:100	Threshold at 5% positive nuclei
Progesterone receptor	Lab Vision, SP2	0.01 M citrate NaOH, pH 6.0	1:2,000	Threshold at 5% positive nuclei
HER2/*neu *immunohistochemistry	Dako, polyclonal	0.01 M citrate NaOH, pH 6.0	1:200	0 - 1 + -2 + 3 + according to DAKO manual
Cytokeratin 5/6	Dako, D5/6 B4	0.001 M EDTA-NaOH, pH 8.0	1:200	Threshold at 1% positive tumor cells
Cytokeratin 14	Novocastra, LL002	0.01 M citrate NaOH, pH 6.0	1:200	Threshold at 1% positive tumor cells
Cyclin D_1 _immunohistochemistry	Lab Vision, SP4	0.001 M EDTA-NaOH, pH 8.0	1:800	Threshold at 5% positive nuclei
Mucin 1	Novocastra, MA695	0.001 M EDTA-NaOH, pH 8.0	1:800	0 negative; 1, 1% to 50%; 2, >50% tumor cells positive
Gross cystic fluid protein	Novocastra, 23A3	0.001 M EDTA-NaOH, pH 8.0	1:800	Threshold at 1% positive tumor cells

*In situ *hybridization	Probe	Protocol	Evaluation	

HER2/*neu *amplification	Abbott PathVysion HER2 DNA Probe Kit	Manufacturer's instructions	Locus-specific signal to centromere signal ratio >2.0 considered amplified	
Cyclin D_1 _amplification	Abbott Vysis LSI Cyclin D1/CEP11	Manufacturer's instructions	Locus-specific signal to centromere signal ratio >2.0 considered amplified	

EDTA, ethylenediamine tetraacetic acid. Lab Vision (Thermo Fisher Scientific, Fremont CA, USA); Novocastra (Leica Microsystems, Wetzlar, Germany); Dako (Glostrup, Denmark); Abbott (Abbott Park, IL, USA).

### Sample processing

The lumpectomy or mastectomy resection specimens were received within 20 minutes of surgical removal and were immediately evaluated by a pathologist. Tissue pieces of approximately 3 mm × 3 mm × 8 mm were cut from redundant tumor tissue after standard surgicopathological processing, were snap-frozen in liquid nitrogen and were stored at -80°C. All samples analyzed were stored for no longer than 2 years, and were thawed once immediately before analysis using SELDI-TOF MS in January 2006.

The tissue microarrays were constructed from routinely prepared formalin-fixed paraffin-embedded tissue blocks taken in parallel, using manual tissue arrayer TA1 (A Fintajsl, Czech Republic). Tissue lysis was performed in guanidine buffer (100 mM phosphate buffer, pH 6.6; 6 M guanidine–HCl, 1% Triton X-100) [23] with vigorous shaking for 1 hour at room temperature followed by centrifugation for 30 minutes at 14,000 × *g*. The total protein concentration was measured by Bradford assay (Bio-Rad, Hercules, CA, USA), and the lysates were aliquoted and stored at -80°C.

### SELDI-TOF MS analysis

A pilot study was performed using IMAC30 chips, CM10 (with cation exchange surface, buffer pH 4) and Q10 arrays (with anion exchange surface, buffer pH 9). The final selection was made on the basis of number of peaks detected in spectra (IMAC30 and CM10 displayed a similar number of peaks, Q10 displayed about one-half the number of peaks), on the overall intensity of spectra (the IMAC30 has higher intensity than CM10) and in agreement with published results of other studies previously performed with breast tissue lysates using IMAC30 arrays [17,21].

Protein profiling was performed on IMAC30 ProteinChip Arrays (Bio-Rad) preactivated with copper and following the manufacturer's instructions. A total of 20 μg protein was applied to each spot, diluted 16 times in binding buffer. Finally, 2 × 1 μl sinapinic acid (saturated solution in 0.5% trifluoroacetic acid/50% acetonitrile) was applied. Chips were analyzed on the ProteinChip Reader Model PBS IIc (Bio-Rad) in positive linear mode. Each sample was applied randomly and measured twice. The laser intensity was set at 190 (in relative units, with a range from 0 to 300), detector sensitivity at 5 (in relative units, within the range of 1 to 10) and focus mass at 10 kDa to obtain mass data within the range of 3 to 100 kDa. Each spot (divided into 100 positions) was measured from positions 20 to 80 with steps of four positions. At each single position, 13 transients resulting in 195 shots per spot were measured.

All spectra were collected together into one experimental file. Two spectra, which did not contain any peaks in consequence of an array processing error, were eliminated. To ensure the mass accuracy, all spectra were calibrated using external mass standards: hirudin BHVK (6,964 Da), equine myoglobin (cardiac, 16,951.5 Da), and bovine carbonic anhydrase RBC (29,023.7 Da). To avoid interspectra variability, the intensities of all spectra were normalized to the total ion current using an external normalization coefficient (Ciphergen ProteinChip software 3.2.1; Bio-Rad, Hercules, CA, USA). The value of this coefficient was set at 0.09 (the total ion current value of the least intensive spectrum) to keep the normalization factor of all spectra no higher than one. The normalization process takes the total ion current used for all the spots, averages the intensity and adjusts the intensity scales for all the spots, enabling one to display all the data on the same scale. The baseline ion current values were automatically subtracted to calculate the total ion current measurements; the spectra were normalized in a range from 2 to 100 kDa. Molecular masses below 2 kDa were not analyzed due to masking of peptide signals by peaks from the sinapinic acid matrix.

Finally, we performed internal mass calibration using an endogenous peak at 15,841 Da that appears in all the selected spectra. This step adjusts the mass scaling (*x *axis) of the entire spectrum on the basis of the naturally occurring sample peaks. External calibration was performed once before the study and was controlled during the study period without any significant changes. Next, peak clustering was calculated with Biomarker Wizard software (Bio-Rad, Hercules, CA, USA) with a signal/noise ratio >5 and 5% minimum spectra detection in the first pass, and then peaks with a signal/noise ratio >3 in a cluster mass window of range 0.3% were added; the valley depth was set at three times the noise. Peak intensities from duplicate samples were then averaged. Ultimately, a final set of 130 identified peak clusters was used for analysis.

### Statistical analysis of SELDI-TOF MS mass spectra

The dataset comprised protein expression profiles of 105 samples, each represented by 130 peaks. Together with expression profiles, additional information about 17 clinicopathological variables was available. We performed unsupervised clustering of carcinoma cases according to their protein expression profiles, and we tested distribution of categories of clinical and molecular variables in these groups.

To reveal subgroups of peaks and patients, the dataset was analyzed by hierarchical clustering with the Ward method of linkage of clusters [24] and the distance measure was derived from the Spearman correlation matrix. The Ward method attempts to minimize the sum of squares of any two (hypothetical) clusters that can be formed at each step. The distance measure was derived as absolute value of (Spearman correlation-1). The maximum distance of two therefore represents a Spearman correlation of -1, and the minimal distance of zero represents a Spearman correlation of 1.

For analysis of the relationship between categorical clinicopathological variables and the different groups of women (as estimated by cluster analysis), a Fisher exact test (for 2 × 2 contingency tables) and a maximum likelihood chi-square test (for *n *× *n *contingency tables) were performed. Kruskall–Wallis analysis of variance was used to compare the age distribution between groups. All hypotheses were tested at significance level α = 0.05. To avoid a multiple testing problem, the α value was adjusted by a Bonferroni correction for the appropriate number of clinicopathological variables, thus obtaining α = 0.05/17 = 0.0029.

The parameters were tested against three main clusters (A, B, C) and against five smaller clusters (I, I, II, IV, V). For some parameters, dividing patients into five clusters resulted in relatively low numbers in some categories; it is therefore not possible to draw any general conclusions on the results of these parameters, but these *P *values are included for informative purposes.

Multivariate analysis was performed using R statistical software version 2.4.0 (R Development Core Team, R foundation for statistical computing, Vienna, Austria). Statistical testing was performed using the STATISTICA 7.1 software (StatSoft Inc., Tulsa, OK, USA).

### Protein identification

Tissue lysate was preseparated using four IMAC Spin Columns (Bio-Rad Laboratories, Hercules, CA, USA) (1 mg protein lysate for each column) according to the manufacturer's instructions, with 2 × 100 μl of 250 mM imidazole in binding buffer as the elution buffer. Eluted protein mixtures were dialyzed overnight against 40 mM Tris–HCl buffer (pH 7.0), combined and dried under vacuum. The pellet of IMAC preseparated proteins was resolubilized in 20 μl reducing sample buffer A [25] for tricine SDS-PAGE (see below), or in 80 μl sample solution B containing 10% acetonitrile in 0.1% trifluoroacetic acid for further reverse phase-liquid chromatography fractionation. This fractionation was performed on an Agilent HP 1100 HPLC system (Agilent Technologies, Santa Clara, CA, USA) using a Discovery Bio Wide Pore C18 column (10 cm × 2.1 mm, 5 μm particle size; Sigma-Aldrich Corp., St. Louis, MO, USA) with a 2 cm guard precolumn. Separations were performed at 35°C, and mobile phase A consisted of 0.1% trifluoroacetic acid in water while mobile phase B consisted of 0.1% trifluoroacetic acid in acetonitrile.

The proteins were eluted using a linear gradient of mobile phase B (0% to 100% B in 25 minutes) followed by elution using 100% mobile phase B in 10 minutes; the flow rate was 100 μl/min (following the method of Moshkovskii and colleagues [25], with several modifications). The collected fractions (60 μl each) were dried under vacuum and resolubilized in 20 μl sample solution B for protein profile determination on SELDI-TOF MS (2 μl each resolubilized fraction was analyzed on NP20 chips). Selected fractions containing the protein(s) of interest were mixed together and dried under vacuum. The proteins were redissolved in 20 μl sample buffer A, heated (95°C/3 minutes), centrifuged (16,000 × *g*/20 minutes/4°C) and separated using tricine SDS-PAGE on PROTEAN II XL apparatus (Bio-Rad) according to Schagger [26].

The gel – consisting of 4% sample gel, 10% spacer gel and 16% separation gel – was stained using colloidal Commassie Blue. The bands with appropriate molecular weight were cut out and digested by trypsin according to Havlasova and colleagues [27]. Mass spectra were acquired using a 4800 MALDI TOF/TOF™ spectrometer (Applied Biosystems, Foster City, CA, USA) in both positive reflectron and MS/MS modes. GPS Explorer™ software (version 3.6; Applied Biosystems, Foster City, CA, USA) was used for the evaluation of mass spectra and the identification of proteins.

## Results

**Figure 1:**
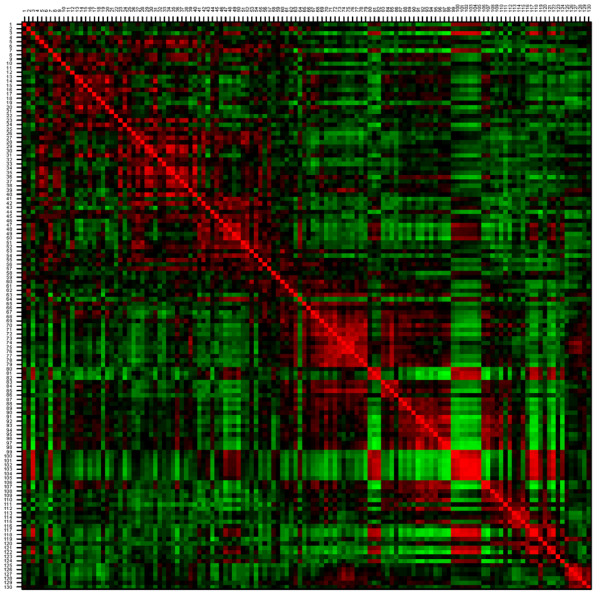
Graphical representation of Spearman correlation matrix of 130 surface-enhanced laser desorption/ionization time-of-flight mass spectrometry peaks. Red color intensity, positive correlation; green color intensity, negative correlation.

IMAC 30 (immobilized metal affinity capture) ProteinChip arrays were used to analyze tissue lysates from 105 breast cancer patients. After data processing by Biomarker Wizard software, a total of 130 peaks were selected. Information about the peaks is presented in Additional file 1. The normalized linear intensities of peaks analyzed by hierarchical clustering revealed subgroups of peaks and of patients. The graphical representation of Spearman correlation matrix of peaks is shown in Figure 1. These data clearly demonstrate the groups of peaks that are highly positively correlated, indicating coexpression of these peaks in individual tumors. The hierarchical clustering combines peaks into two, three, or six potential groups, respectively, according to their level of positive correlation. In each of these categories we can find groups of adjacent correlated peaks, as apparent in Figure 1. The highest correlation can be found between peaks from 80 to 82, peaks from 99 to 105, 117 and 118, and peaks from 121 to 124, which form the first group of six categorizations. Descriptive statistics for peaks classified into the six groups are summarized in Table 2. Note that the groups are listed according to decreasing minimal correlation within the group. Using the Spearman correlation matrix to derive the distance matrix for hierarchical clustering, the categorization of patients was most strongly affected by groups of highly correlated peaks.

**Table 2:** Descriptive statistics of groups of peaks as revealed by hierarchical clustering

Categorization			Spearman correlation between peaks in the category							
			
			*N*	Median	Mean	Minimum	Maximum	% < 0.1	% 0.1 to 0.5	% > 0.5
1	1	1	120	0.76	0.74	0.35	0.99	0.0	8.3	91.7
		2	153	0.44	0.44	0.02	0.83	0.0	60.1	39.9
2	2	3	120	0.34	0.34	-0.14	0.94	0.0	50.1	33.2
		4	378	0.26	0.28	-0.26	0.92	5.6	74.6	19.8
	3	5	210	0.43	0.38	-0.29	0.96	5.2	57.6	37.1
		6	465	0.29	0.28	-0.42	0.90	11.0	68.0	21.1

% < 0.1, percentage of correlations with value smaller than 0.1; % 0.1 to 0.5, percentage of correlations in the interval 0.1 to 0.5; % > 0.5, percentage of correlations with values greater than 0.5. Categories are ordered according to decreasing minimal correlation in the group. *N*, number of correlations between peaks in the category: *N *= (number of peaks in the category^2 ^- number of peaks in the category)/2).

**Figure 2:**
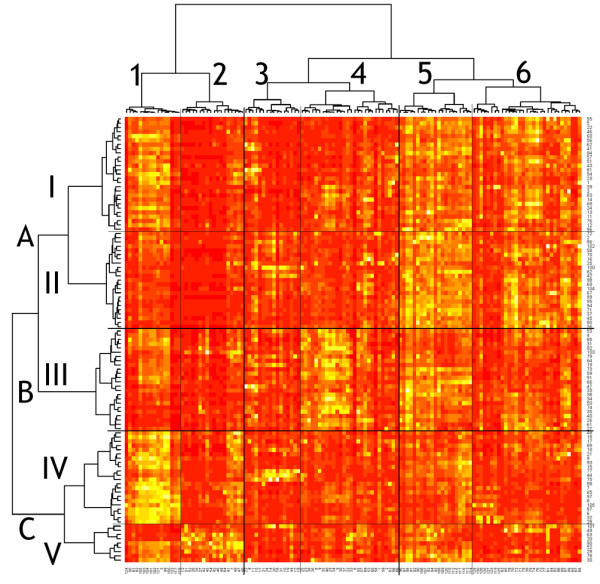
Result of hierarchical clustering in the form of a heat map of peak values. Rows represent 105 individual patients and columns represent 130 peaks used for the analysis. The value of the peak is indicated by the color intensity. Unsupervised hierarchical clustering revealed two (not labeled), three (labeled A, B, C), five (labeled I to V) and six (labeled 1 to 6) groups of patients.

Figure 2 shows the result of hierarchical clustering in the form of a heatmap of values of peaks, where rows represent individual patients and each column represents one of the 130 peaks used for the analysis. Unsupervised hierarchical clustering of patients (Figure 2) revealed two, three and five potential groups. Categorization into five groups of patients (details in Additional file 2) and into six groups of peaks (details in Additional file 3) shows that, for the first and the second groups of patients, high values of the fifth and sixth groups of peaks are characteristic, while the first group reveals also higher values in the first cluster of peaks. The third group of patients is characterized by higher values in the third cluster of peaks, especially peaks between 1 and 9 and between peaks 23 and 40. The fourth group of patients reveals very high values in the first cluster of peaks, and the fifth group of patients is mainly determined by high values in the second cluster of peaks.

**Figure 3:**
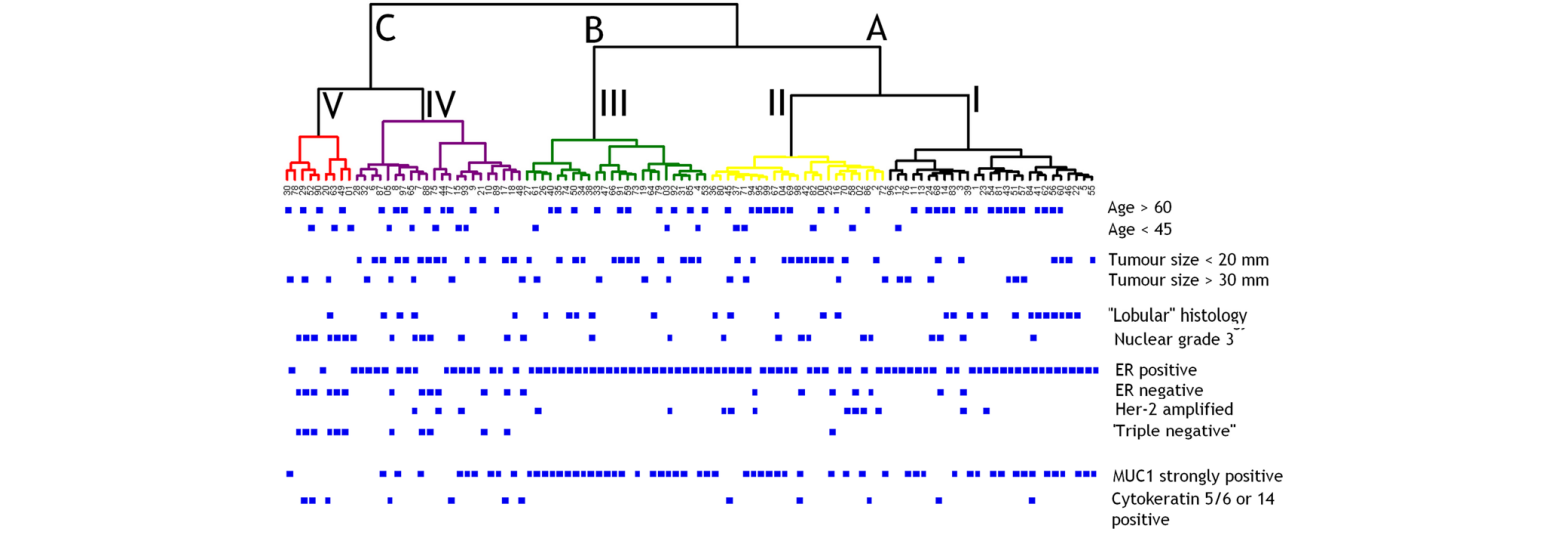
Distribution of selected clinicopathological parameters within the cluster tree of patients. Each square label represents a case. ER, estrogen receptor; MUC1, mucin 1.

The relative frequencies of selected clinicopathological parameters are illustrated in Figure 3 and a complete distribution of clinical variables and molecular markers within the groups of patients is presented in Table 3. The 105 cases are separated into three main clusters (A, B, C) or into five smaller clusters (I to V), significantly associated with tumor type, ER status and nuclear grade. The older patients with tumors expressing hormonal receptors and a high level of mucin 1 tend to group into clusters I to III (or A and B), while the carcinomas of younger patients exhibiting triple negative (that is, ER, progesterone receptor, HER2/*neu*) phenotype, high nuclear grade and basal cytokeratin 5/6 and/or cytokeratin 14 locate more often to cluster IV and especially cluster V (or C).

**Table 3:** Distribution of clinicopathological variables within groups of patients as revealed by hierarchical clustering

		Clusters of patients^a^				
		
		A		B	C	
		
		I	II	III	IV	V
Distribution	Count	27	23	24	22	9
Age	Mean	64.3	58.3	59.6	55.7	55.4
*p*^5 ^= 0.235, *p*^3 ^= 0.212, *N* = 105	Median	64	55	60	57	49
Tumor type	Lobular and mixed ductal/lobular	12	5	5	4	1
*p*^5 ^= 0.040*, *p*^3 ^= 0.021*, *N* = 105	Ductal not otherwise specified	15	17	16	14	4
	Mucinous, papillary	0	0	2	2	1
	Medullary, spindle cell	0	1	1	2	3
Lymph node metastases	Absent	10	8	6	12	5
*p*^5 ^= 0.240, *p*^3 ^= 0.065, *N* = 105	Present	17	15	18	10	4
Maximal tumor diameter	<20 mm (pT1)	6	11	12	13	0
*p*^5 ^= 0.001*, *p*^3 ^= 0.408, *N* = 105	> 20 mm (pT2, pT3)	21	12	12	9	9
Tumor grade	Grade 1	8	5	7	7	2
*p*^5 ^= 0.138, *p*^3 ^= 0.199, *N* = 105	Grade 2	14	7	10	5	1
	Grade 3	5	11	7	10	6
Nuclear grade	Grade 1	6	2	2	6	2
*p*^5 ^< 0.001**, *p*^3 ^< 0.001**, *N* = 105	Grade 2	17	15	20	9	0
	Grade 3	4	6	2	7	7
Estrogen receptor alpha	Positive	25	18	24	15	3
*p*^5 ^< 0.001**, *p*^3 ^< 0.001**, *N* = 105	Negative	2	5	0	7	6
Estrogen receptor beta	Positive	20	11	13	8	3
*p*^5 ^= 0.078, *p*^3 ^= 0.030*, *N* = 99	Negative	7	8	10	13	6
Progesterone receptor	Positive	23	16	24	14	4
*p*^5 ^< 0.001**, *p*^3 ^< 0.001**, *N* = 105	Negative	4	7	0	8	5
HER2 amplification by fluorescence *in situ *hybridization	Absent	25	16	22	19	9
*p*^5 ^= 0.071, *p*^3 ^= 0.398, *N* = 105	Present	2	7	2	3	0
HER2/*neu *by immunohistochemistry	3+	2	5	2	3	0
*p*^5 ^= 0.008*, *p*^3 ^= 0. 046*, *N* = 105	2+	0	3	3	2	0
	1+	10	10	7	2	1
	0	15	5	12	15	8
Cytokeratin 5/6 or cytokeratin 14	Positive	2	3	0	4	3
*p*^5 ^= 0.035*, *p*^3 ^= 0.010*, *N* = 99	Negative	24	18	23	16	6
Triple-negative phenotype	Yes	0	1	0	5	6
*p*^5 ^< 0.001**, *p*^3 ^< 0.001**, *N* = 105	No	27	22	24	17	3
Cyclin D_1 _amplification by fluorescence *in situ *hybridization	Absent	22	21	18	17	9
*p*^5 ^= 0.354, *p*^3 ^= 0.875, *N* = 102	Present	5	2	4	4	0
Cyclin D_1 _by immunohistochemistry	Positive	26	17	21	18	4
*p*^5 ^= 0.001**, *p*^3 ^= 0.111, *N* = 101	Negative	0	5	2	3	5
Mucin 1	2+	17	12	21	9	1
*p*^5 ^= 0.001**, *p*^3 ^< 0.001**, *N* = 102	1+	9	10	2	7	7
	0	1	0	0	5	1
Gross cystic fluid protein	Positive	10	10	13	9	2
*p*^5 ^= 0.572, *p*^3 ^= 0.440, *N* = 98	Negative	15	13	10	10	6

*p*^5 ^values, results of statistical testing within five groups of patients and are rather informative because of the small number of patients in each group; *p*^3 ^values, result of statistical testing within three groups of patients. *P values significant at the 5% significance level. **P values significant at a significance level adjusted by Bonferroni correction (0.05/17 = 0.0029). For some parameters evaluated in tissue microarrays, information was not available in all patients. ^a^A to C, three clusters; I to V, five clusters.

Clusters I and III differ in relative frequency of lobular carcinomas (predominate in cluster I) and ductal carcinomas (predominate in cluster III), otherwise sharing similar characteristics (ER-positive, low grade, older patients). Cluster II is characterized with higher nuclear and tumor grade if compared with adjacent clusters I and III, and contains one-half of the 14 cases exhibiting HER2/*neu *gene amplification. Cluster IV exhibits some transitional characteristics from clusters I to III to cluster V, where the high-grade, triple-negative carcinomas with low expression of mucin 1 and gross cystic fluid protein clearly predominate. Cyclin D_1 _coding gene amplification is randomly distributed except in cluster V. Cyclin D_1 _protein expression is distributed similarly to ER, and the same applies for ERβ. The distribution of lymph node metastases does not exhibit a specific relationship with clustering.

Clustering of patients into five groups was determined by the expression profile of all 130 peaks. To identify these peaks we separated the IMAC binding proteins either by HPLC and tricine SDS-PAGE or directly using tricine SDS-PAGE with subsequent MS/MS identification. For the final identification we extracted four gel bands with identical SELDI-TOF MS and tricine gel molecular masses (7,706 Da, 17,599 Da, 27,152 Da and 33,327 Da corresponding to peaks 81, 107, 114 and 119). Using MS/MS analysis of samples separated by reverse phase-liquid chromatography prior to tricine SDS-PAGE, we identified a peak with molecular mass of 33 kDa as annexin 5 (accession number NP 001145). MS/MS analysis of IMAC binding proteins directly separated by tricine SDS-PAGE lead to identification of a peak with mass 27 kDa as heat shock protein 27 (hsp27) (accession number AAA62175). Both proteins were identified with a protein score confidence interval of 100%. The result of identification of two remaining peaks was not successful.

## Discussion

The goal of expression profiling of human tumors is to provide information that can assist with tumor diagnosis or classification, or can provide prognostic information. To be of value, such data must improve on the clinicopathological assessments already used. Gene expression profiling has shown its worth in these respects in recent years, and in breast cancer has provided alternative classification systems that appear valuable in clinical practice. Protein expression profiling has also been useful in studies of human tumors and has often been used to identify serum biomarkers that aid disease monitoring and the therapeutic response. In the present article we analyzed protein profiles in breast cancers and evaluated the potential for this approach to provide clinically useful classifications of this heterogeneous disease.

The protein expression profiles of tumors obtained in our study, consisting of as little as 130 substantially intercorrelated peaks, must certainly represent only a small fraction of tumor nature. The diversity in profiles reflects necessarily both biological diversity and methodological factors, including variable tumor/stroma ratios. Nonetheless, our data clearly show that this approach provides robust, statistically significant groupings of patients that are related to the recently described classifications based on cDNA expression profiling. Correlating protein expression patterns to tumor morphology, established biomarkers and clinical outcomes is therefore a key issue of high-throughput studies to discover peaks that merit further investigation.

The categories of patients identified by hierarchical clustering of SELDI-TOF MS peaks resemble the recently adopted classifications of breast carcinomas based on gene expression profiling [2]. In this study the first subgroup, so-called luminal subtypes (A and B), makes up the hormone receptor-expressing breast cancer and has expression patterns reminiscent of the luminal epithelial component of the breast (including luminal cytokeratin 8/18 and cyclin D_1 _genes). The second subgroup is called the HER2/*neu *subtype, but should not be confused with HER2/*neu*-positive tumors identified by immunohistochemistry or fluorescence *in situ *hybridization because not all clinically HER2/*neu*-positive tumors show the RNA expression changes that define this subtype. Tumors that do not express hormone receptors are included in this group. The last group of breast cancers distinguished by gene expression profiling is called basal-like breast cancer and lacks expression of ER, has low expression of HER2/*neu*, has strong expression of basal cytokeratins 5, 6 and 14, and expresses high levels of proliferation-related genes [4-6,28].

Our groups I and III exhibit striking similarity to luminal A tumors, group II resembles luminal B carcinomas, while groups IV and V resemble HER2/*neu *subtype and basal carcinomas. On the other hand, we found no significant relationship of our tumor clusters to the presence of lymph node metastases. Regarding the potential prognostic significance of our data, the follow up of the patients is not yet long enough to allow a valuable analysis.

Although controversial, it may be acceptable to use a proteomic polypeptide profile consisting of as yet unsequenced peptides for the diagnosis of disease and to predict the risk of disease development and progression and/or the efficacy of treatment – if it has proven its value in blinded multicenter and repeated studies, as recently pointed out [29]. For a fuller understanding of disease processes and to simplify the investigative procedures, it may also be useful to identify the biomarkers. We were able to identify peak 114 from the fifth peak cluster group with mass 27,152 Da as hsp27. This protein is a molecular chaperone, participating in cell homeostasis under stress conditions, and is associated with resistance of tumors to chemotherapeutics, radiation and hyperthermia [30].

High intensities of all peaks occurring in fifth and sixth peak cluster groups, including the hsp27 peak, classify patients into subcohorts I and III corresponding to luminal A subtype of tumors with high expression of ER. This finding is in agreement with Ciocca and colleagues, who showed correlation of high levels of hsp27 with ER expression [31]. Recent studies revealed the correlation of hsp27 phosphorylation status (that also increases protein binding on the IMAC surface) with HER2/*neu *and lymph node positivity in breast cancer [32].

We could also identify the peak at 33,327 Da from the sixth peak cluster group as annexin 5. The annexin family has been linked to inhibition of phospholipase activity, exocytosis and endocytosis, signal transduction, organization of the extracellular matrix, resistance to reactive oxygen species and DNA replication [33]. Annexin 5 normally forms a shield around certain phospholipid molecules that blocks their entry into coagulation reactions [34]. The role of annexin 5 in breast cancer, however, is elusive.

Our methodological approach of unsupervised hierarchical clustering helped overcome some of the complexities of SELDI-TOF MS to yield biologically meaningful and interpretable data. In the present study we demonstrated that, using the whole protein spectra, we were able to cluster our patients into subcohorts paralleling classification based on DNA microarray profiling data.

## Conclusion

Our results show that SELDI-TOF MS protein profiling distinguishes between different groups of primary human breast cancers and produces a similar clustering of tumors as cDNA expression profiles. This fact testifies to the validity of the SELDI-TOF MS proteomic approach in these types of study. As SELDI-TOF MS provides different information compared with cDNA expression profiles, the results suggest its potential to supplement and expand our knowledge of breast carcinomas. The identification of proteins belonging to the interesting peaks followed by validation by immunohistochemistry is an essential task for future studies. Although many parameters have been explored in relation to breast cancer biology and outcome, the ability to classify tumors into distinct subclasses by identifying representative protein expression patterns improves our understanding of cancer biology and provides the potential for more precise clinicopathological phenotyping.

## Abbreviations

ER = estrogen receptor; HPLC = high-performance liquid chromatography; SELDI-TOF MS = surface-enhanced laser desorption/ionization time-of-flight mass spectrometry.

## Competing interests

The authors declare that they have no competing interests.

## Authors' contributions

KB performed the laboratory work for SELDI-TOF MS analysis of tissue lysates, analyzed the mass spectra obtained from this analysis, participated in protein identifications and helped to draft the manuscript. EB performed the statistical analysis and helped to draft the manuscript. PB performed in part the analysis of SELDI-TOF MS data, performed the protein separations for MS identification and helped with interpretation of the data. LH performed MS analysis and data interpretation. DK prepared all of the tissue samples for SELDI-TOF MS. DV supervised and reviewed the SELDI-TOF MS analysis. RV participated in the study design and supervision of clinical data analysis. BV participated in the initiation of the project and study design, financially supported the project and helped to draft the manuscript. RN conceived the study, participated on interpretation of the results and statistical analysis, supervised the statistical analysis and finalized the manuscript. All authors read and approved the final manuscript.

## Supplementary Material

Additional file 1 A table that provides information about peaks and their masses. Click here for file

Additional file 2 A table that provides identification of patients within patient groups. Click here for file

Additional file 3 A table that provides identification of peaks within peak groups. Click here for file
