# Supplementary material for: Surface-enhanced laser desorption/ionization time-of-flight proteomic profiling of breast carcinomas identifies clinicopathologically relevant groups of patients similar to previously defined clusters from cDNA expression
Source: Breast Cancer Res. 2008 May 29;10(3):R48. doi: 10.1186/bcr2101 (PMC2481497; doi:10.1186/bcr2101)
Supplement: Additional file 2 — A table that provides identification of patients within patient groups. [file bcr2101-S2.pdf]

SUPPLEMENTARY INFORMATION Brozkova et al. Table C

Classification of patients according to hierarchical clustering.

<table>
<tr><th colspan="3">Categorization</th><th>Patients in the group</th></tr>
<tr><td rowspan="3">1</td><td rowspan="2">A</td><td>I</td><td>1,3,5,11,12,13,14,22,23,24,39,41,43,46,51,54,55,56,57,60,62,68,76,81,83,84,96</td></tr>
<tr><td>II</td><td>2,16,25,36,37,42,45,58,67,69,70,71,72,80,82,86,94,95,98,99,100,102,104</td></tr>
<tr><td>B</td><td>III</td><td>4,19,26,27,31,33,34,35,38,40,47,50,53,59,61,64,66,73,74,79,85,91,92,103</td></tr>
<tr><td rowspan="2">2</td><td rowspan="2">C</td><td>IV</td><td>6,7,8,9,10,15,17,18,21,28,32,44,48,65,75,77,87,88,89,93,97,105</td></tr>
<tr><td>V</td><td>20,29,30,49,52,63,78,90,101</td></tr>
</table>
