# Supplementary material for: Surface-enhanced laser desorption/ionization time-of-flight proteomic profiling of breast carcinomas identifies clinicopathologically relevant groups of patients similar to previously defined clusters from cDNA expression
Source: Breast Cancer Res. 2008 May 29;10(3):R48. doi: 10.1186/bcr2101 (PMC2481497; doi:10.1186/bcr2101)
Supplement: Additional file 3 — A table that provides identification of peaks within peak groups. [file bcr2101-S3.pdf]

SUPPLEMENTARY INFORMATION Brozkova et al. Table B

Table B. Classification of peaks according to hierarchical clustering.

<table>
<tr><th colspan="3">Categorization</th><th>Peaks in a group</th></tr>
<tr><td rowspan="2">1</td><td rowspan="2">1</td><td>1</td><td>3 80,81,82 99,100,101,102,103,104,105 117,118 121,122,124</td></tr>
<tr><td>2</td><td>7 12 26,27,28 41,42,43,45,46,47,48,49,50,51,52,54 64</td></tr>
<tr><td rowspan="4">2</td><td rowspan="2">2</td><td>3</td><td>4 10,11,13,14,15,16,17,18,19,20,21,22,24 31 44</td></tr>
<tr><td>4</td><td>1,2,5,6,8,9 23,25 29,30,32,33,34,35,36,37,38,40
53,55,56,57,58,59,60,62 66 69</td></tr>
<tr><td rowspan="2">3</td><td>5</td><td>90,91,92,93,94,95,96,97,98 106,107,108,109,110,111,112,113,114,115,116 120</td></tr>
<tr><td>6</td><td>39 61,63,65,67,68,70,71,72,73,74,75,76,77,78,79 83,84,85,86,87,88,89
119 123,125,126,127,128,129,130</td></tr>
</table>
